# RECRUITING SAMPLES OF OLDER ADULTS FROM VULNERABLE GROUPS

**DOI:** 10.1093/geroni/igad104.1310

**Published:** 2023-12-21

**Authors:** Lisa Barry

**Affiliations:** UConn Center on Aging, Farmington, Connecticut, United States

## Abstract

Since 2000, the number of incarcerated persons age ≥50 in the U.S. grew 280% and those in this age group with substance use disorders (SUDs) grew from 1.7 to 5.7 million. Yet, these groups, which comprise some of the most medically vulnerable persons in society, are difficult to recruit to research studies. We successfully recruited and retained 171 incarcerated persons age ≥50 in a cohort study. We will discuss how recruitment required strong partnership with the state corrections system, how we had to be cognizant of measures that were meaningful in the context of the unique environment (prison activities of daily living), and how measures were modified (when life gets hard I just want to escape). As we continue to recruit persons ≥50 seeking SUD treatment, we will discuss the usefulness of snowball recruiting and balancing decisions regarding modification of inclusion criteria while maintaining integrity of the original research question.

